# The recombinant pseudorabies virus expressing porcine deltacoronavirus spike protein is safe and effective for mice

**DOI:** 10.1186/s12917-021-03115-1

**Published:** 2022-01-04

**Authors:** Yao Huang, Zhiwen Xu, Sirui Gu, Mincai Nie, Yuling Wang, Jun Zhao, Fengqing Li, Huidan Deng, Jianbo Huang, Xiangang Sun, Ling Zhu

**Affiliations:** 1 College of Veterinary Medicine, Sichuan Agricultural University, Chengdu, 611130 Sichuan China; 2 Key Laboratory of Animal Diseases and Human Health of Sichuan Province, Chengdu, 611130 Sichuan China; 3 College of Animal Science, Xichang University, Xichang, 615000 Sichuan China

**Keywords:** PDCoV, Spike protein, Recombinant pseudorabies virus, Immunogenicity, Protective efficacy

## Abstract

**Background:**

Porcine deltacoronavirus (PDCoV) is a new pathogenic porcine intestinal coronavirus, which has appeared in many countries since 2012. PDCoV disease caused acute diarrhea, vomiting, dehydration and death in piglets, resulted in significant economic loss to the pig industry. However, there is no commercially available vaccine for PDCoV. In this study, we constructed recombinant pseudorabies virus (rPRVXJ-delgE/gI/TK-S) expressing PDCoV spike (S) protein and evaluated its safety and immunogenicity in mice.

**Results:**

The recombinant strain rPRVXJ-delgE/gI/TK-S obtained by CRISPR/Cas gE gene editing technology and homologous recombination technology has genetic stability in baby hamster syrian kidney-21 (BHK-21) cells and is safe to mice. After immunizing mice with rPRVXJ-delgE/gI/TK-S, the expression levels of IFN-γ and IL-4 in peripheral blood of mice were up-regulated, the proliferation of spleen-specific T lymphocytes and the percentage of CD4^+^ and CD8^+^ lymphocytes in mice spleen was increased. rPRVXJ-delgE/gI/TK-S showed good immunogenicity for mice. On the seventh day after booster immunity, PRV gB and PDCoV S specific antibodies were detected in mice, and the antibody level continued to increase, and the neutralizing antibody level reached the maximum at 28 days post- immunization (dpi). The recombinant strain can protect mice with 100% from the challenge of virulent strain (PRV XJ) and accelerate the detoxification of PDCoV in mice.

**Conclusion:**

The recombinant rPRVXJ-delgE/gI/TK-S strain is safe and effective with strong immunogenicity and is expected to be a candidate vaccine against PDCoV and PRV.

**Supplementary Information:**

The online version contains supplementary material available at 10.1186/s12917-021-03115-1.

## Background

Porcine deltacoronavirus (PDCoV) belongs to the family of coronaviruses. The PDCoV genome is a single-stranded, positive-sense RNA of approximately 25 kb in length with the genome organization of δ-CoVs: 5′-UTR-ORF1a/1b-S-E-M-NS6-N-NS7-NS7a-3′UTR [1, 2]. PDCoV was first detected in Hong Kong, China, in 2012 [3] (but not successfully isolated), and subsequently detected in pig farms in the USA, Canada, Korea, China, Thailand, Laos, and Vietnam. PDCoV presents a global distribution trend and is a common pathogen of porcine disease worldwide [4–6]. Typical clinical symptoms of PDCoV infection include diarrhea, dehydration, variable vomiting and mortality in nursing piglets. Similar to that of PEDV and TGEV, the strongest tissue tropism of PDCoV is in villous enterocytes of the small and large intestines, leading to marked villous atrophy in the small intestine but not in the large intestine [7].

PDCoV contains four major structural proteins: spike protein (S), envelope protein (E), membrane protein (M) and nucleocapsid protein (N). Studies of S protein’s structure and function can provide valuable information for assessing the cross-species transmission potential of the virus. The functions of S protein include recognizing receptors, mediating virus into host cells, determining virus host range and tissue tendency, and inducing host immune response [8, 9]. It is the main target of host neutralizing antibody [10].

Pseudorabies virus (PRV) is a α herpes virus can cause high mortality encephalitis in newborn piglets, respiratory diseases and growth retardation in finishing pigs, abortion and stillbirth in sows, has caused huge losses to the world pig industry [11]. The genome of PRV is a linear DNA molecule of about 143 kb, consisting of a unique long region (UL), a unique short region (US), a terminal repeat (TRS), and an internal repeat (IRS) [12]. PRV as a vector can accommodate thousands of bases (kb) of foreign genes. At present, many insertion sites have been identified, such as TK, PK, gE, gI and gG sites [13]. These genes can be deleted or replaced by foreign genes without affecting PRV replication. Therefore, PRV is often used as a vector to express antigenic proteins of other porcine pathogens for the development of multivalent vaccines to protect against porcine pseudorabies and other porcine diseases.

PDCoV and PRV are important infectious virus endangering the global pig industry. They are widespread in many pig farms, which has brought huge economic losses to the pig industry. Vaccine immunization is the most effective ways of disease control. However, there is currently no commercial vaccine available for PDCoV [14]. The vaccine based on recombinant virus has played an important role in the development of new vaccine. In this study, based on rPRVXJ-delTK, the recombinant pseudorabies virus rPRVXJ-delgE/gI/TK-S expressing PDCoV S protein was constructed by using CRISPR/Cas9 gene editing technology and homologous recombination technology, which is expected to highly express protein antigen in host cells. Using the potential adjuvant of the virus delivery system itself, it can be directly and effectively delivered to the immune system to produce cellular immunity and humoral immunity. The safety and the ability to induce humoral and cellular immune responses were evaluated in mice. The results of this study indicate that the recombinant vaccine is a promising candidate vaccine for prevention and control of PDCoV in China.

## Results

### Construction and verification of recombinant virus rPRVXJ-delgE/gI/TK-S

**Fig. 1:**
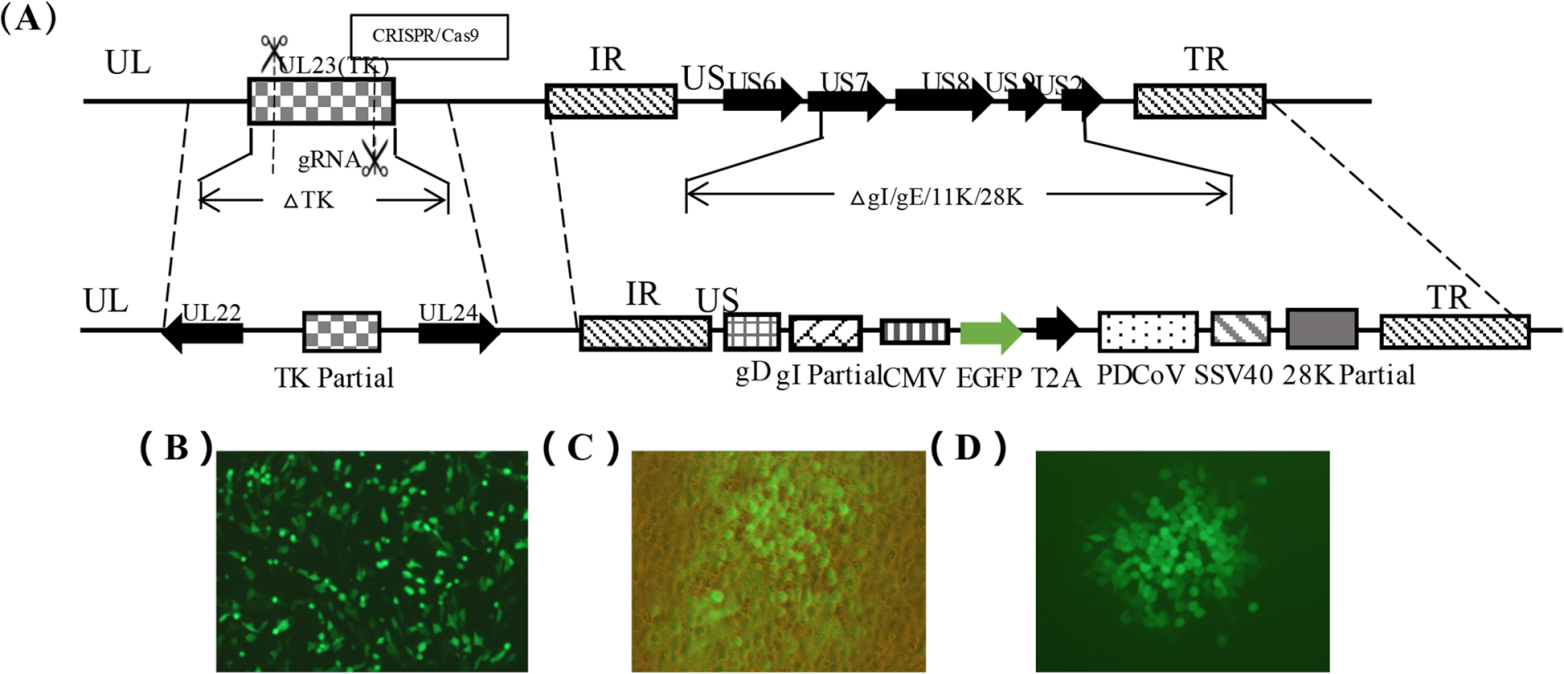
Construction of rPRVXJ-delgE/gI/TK-S. **A**. Schematic diagram of recombinant strain construction. **B**. A large amount of PDCoV S protein was expressed in BHK-21 cell. **C**. rPRVXJ-delgE/gI/TK-S was observed for the first time. D. rPRVXJ-delgE/gI/TK-S after purification

The recombinant pseudorabies virus rPRVXJ-delgE/gI/TK-S expressing PDCoV S protein was constructed by CRISPR/cas9 technology and homologous recombination technology (Fig. 1A). Under the inverted microscope (Nikon, Japan), it can be observed that the green fluorescent labeled protein EGFP is expressed in BHK-21 cells 24 h after transfection (Fig. 1B), the cytopathic effect (CPE) caused by rPRVXJ-delgE/gI/TK-S (Fig. 1C), and the purified recombinant virus (Fig. 1D).

**Fig. 2:**
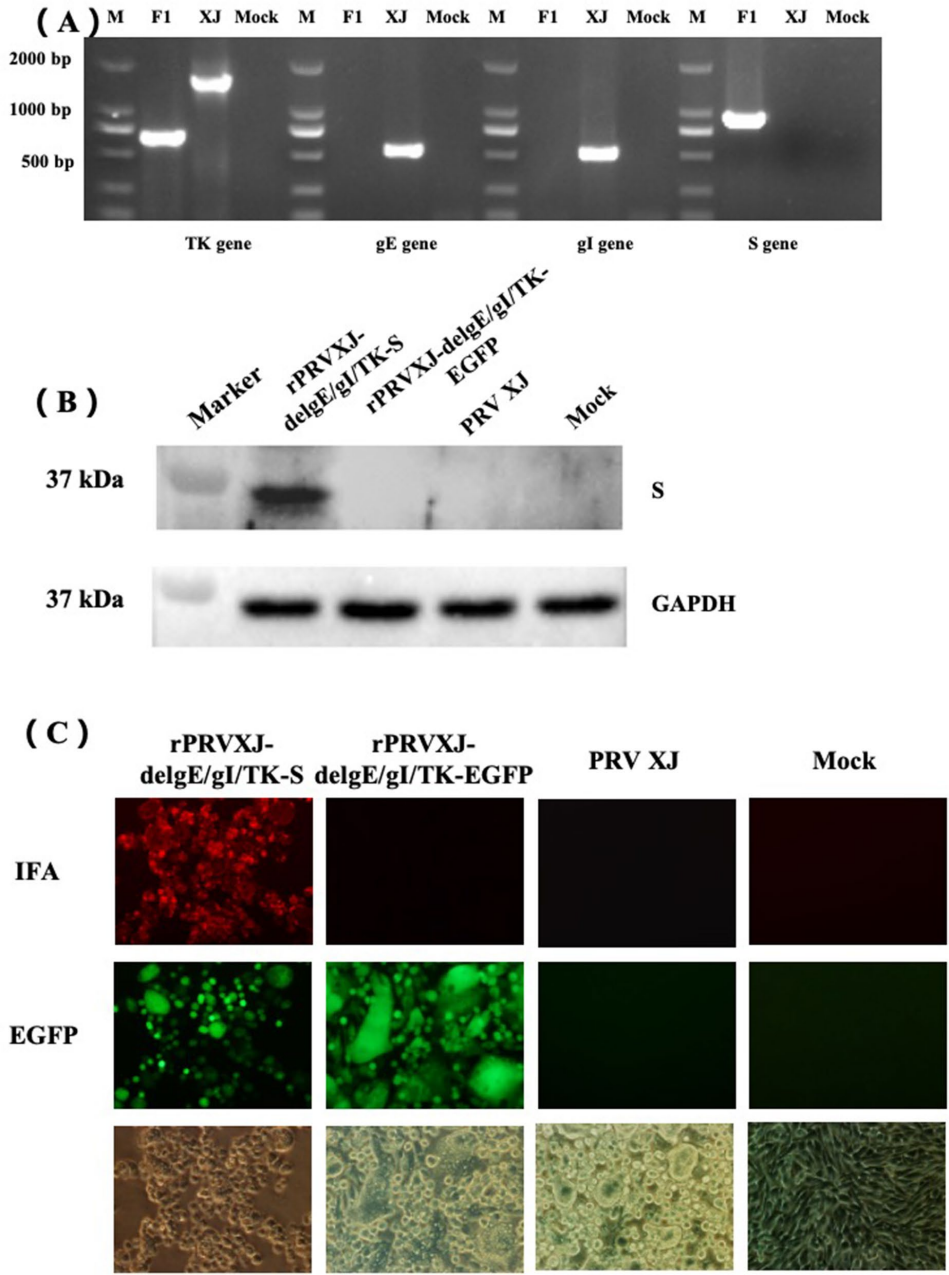
Identification of rPRVXJ delgE/gI/TK-S. **A**. PCR. PCR analysis of deletion (TK, gE and gI genes) / insertion (PDCoV S gene). **B**. Western blotting. The expression of PDCoV S gene in rPRVXJ delgE/gI/TK-S was detected by Western blotting. **C**. Indirect immunofluorescence (IFA). The expression of PDCoV S gene in rPRVXJ delgE/gI/TK-S was detected by indirect immunofluorescence

The deletion of gE, gI and TK genes and insertion of S gene of the recombinant virus were identified by polymerase chain reaction (PCR) (Fig. 2A), PRV XJ DNA was used as the positive control, and the template without DNA was used as the mock control. The expression of S protein was analyzed by western blot and indirect immunofluorescence. Western blot analysis showed that the specific band of S protein (about 34 kDa) could be detected in BHK-21 cells infected with rPRVXJ-delgE/gI/TK-S. However, it was not detected in the control group infected with rPRVXJ-delgE/gI/TK-EGFP, PRV XJ and non-infected group (Fig. 2B). In addition, in indirect immunofluorescence (IFA) analysis, red fluorescence was observed in BHK-21 cells infected with rPRVXJ-delgE/gI/TK-S, but not in the control groups (Fig. 2C).

### Some biological characteristics of recombinant virus rPRVXJ-delgE/gI/TK-S

**Fig. 3:**
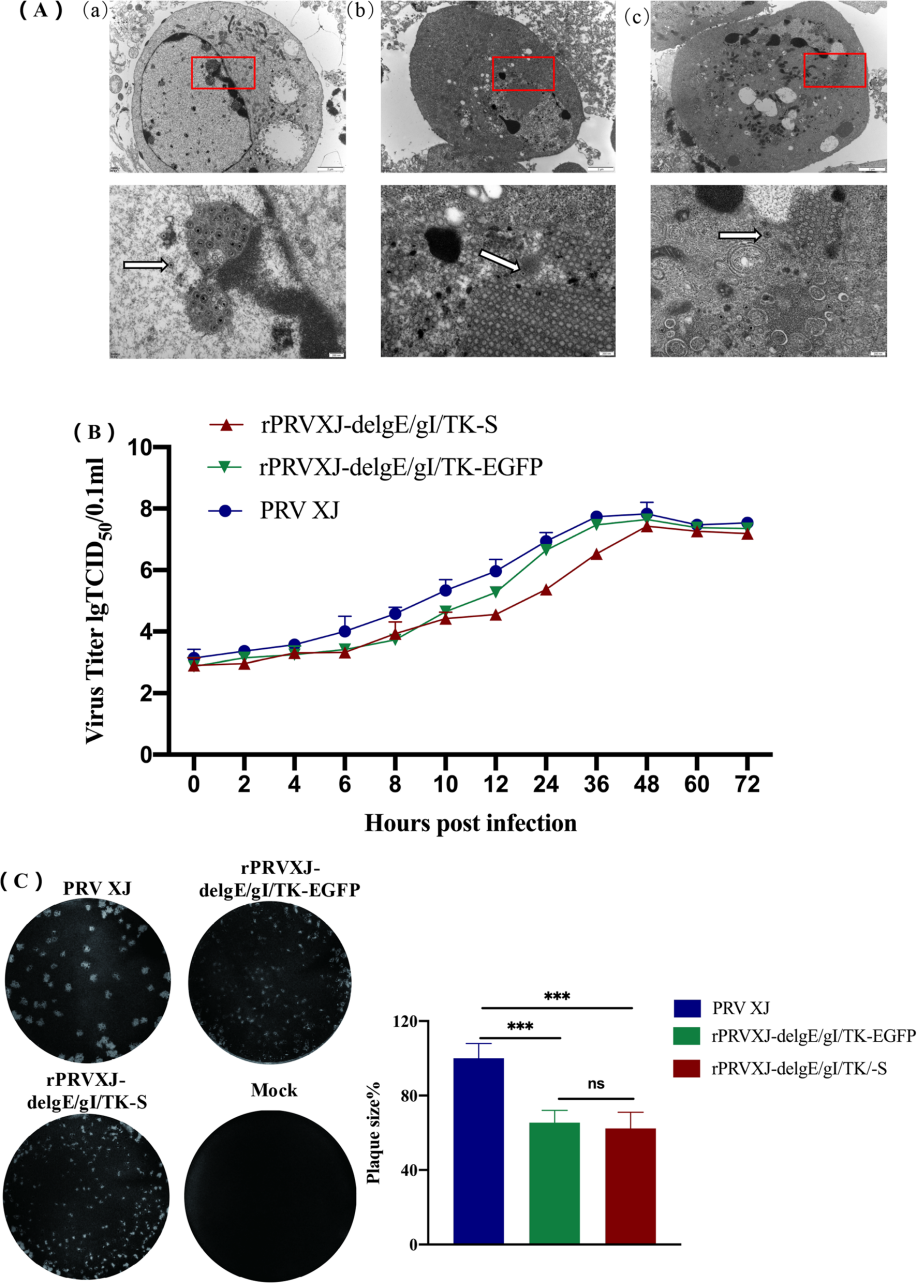
Biological characteristics of rPRVXJ delgE/gI/TK-S**. A**. Electron microscopic observation of recombinant strains. (a) PRV XJ. (b) rPRVXJ-delgE/gI/TK-EGFP. (c) rPRVXJ-delgE/gI/TK-S. **B**. One-step growth curve. **C**. Plaque test. The experiment was repeated three times. Ten plaques were randomly selected for each strain to calculate plaque size. Data are expressed as mean ± SD. Significant difference was indicated by “***” (*P* < 0.001); Extremely significant difference was indicated by “**” (*P* < 0.01); Significant difference was indicated by “*” (*P* < 0.05); No significant difference was indicated by “ns” (*P* > 0.05)

The results of transmission electron microscope showed that the capsid diameter of the recombinant strain and the parent strain was about 110 nm, and there was no size difference (Fig. 3A). The growth characteristics of rPRVXJ-delgE/gI/TK-S, rPRVXJ-delgE/gI/TK-EGFP and their parent strain PRV XJ were compared by BHK-21 cells. The growth dynamics of rPRVXJ-delgE/gI/TK-S and rPRVXJ-delgE/gI/TK-EGFP were similar to those of PRV XJ, but the titers of the recombinant strains were lower than those of the parent strains (Fig. 3B). The plaque size of rPRVXJ-delgE/gI/TK-S and rPRVXJ-delgE/gI/TK-EGFP was the same, but significantly different from that of the parent strain PRV XJ (Fig. 3B).

### Stability of rPRVXJ-delgE/gI/TK-S

**Fig. 4:**
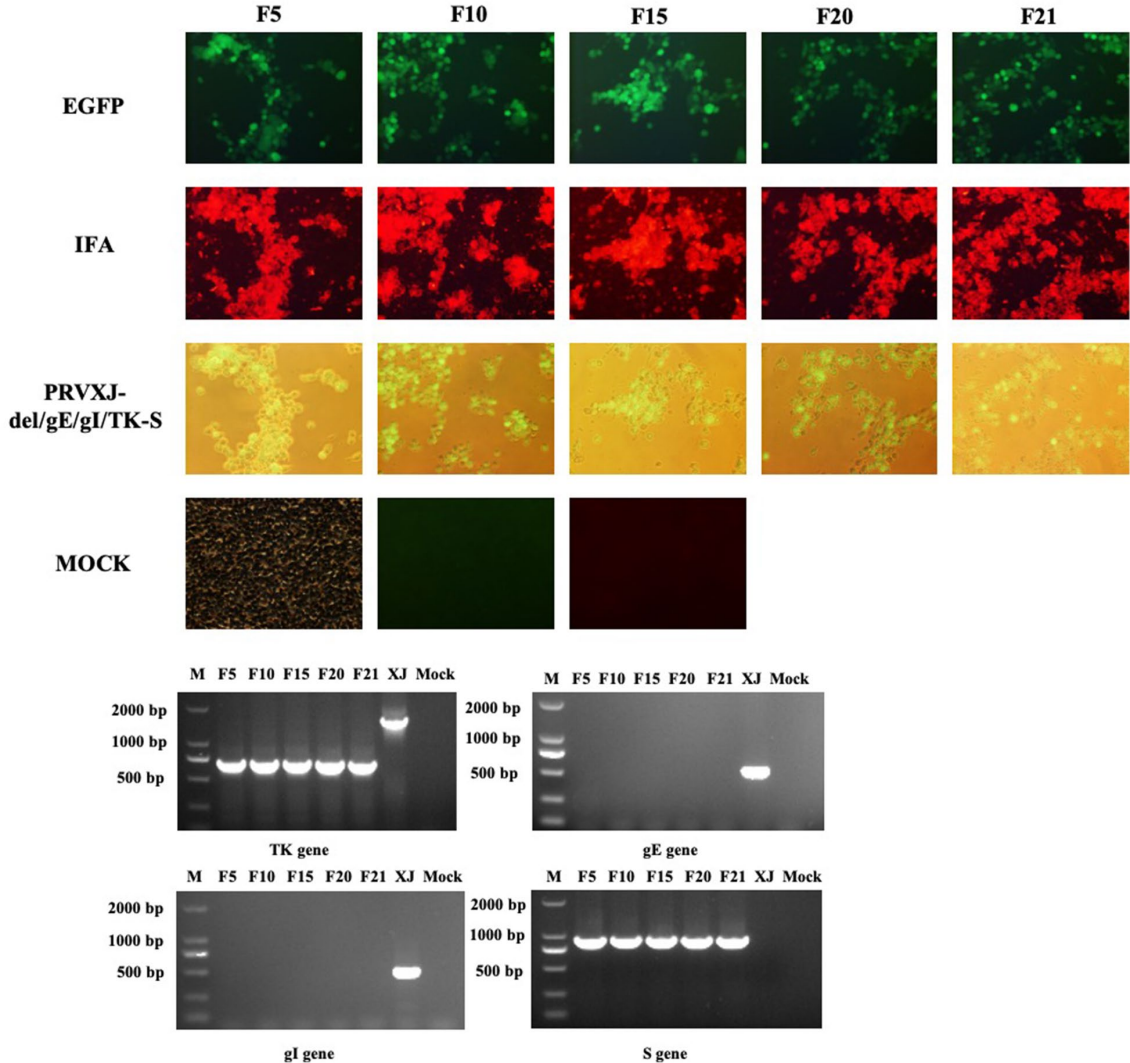
Genetic stability of the rPRVXJ delgE/gI/TK-S. **A**.IFA. The expression of rPRVXJ-delgE/gI/TK-S (F5, F10, F15, F20 and F21) PDCoV S protein was identified by IFA. **B**. PCR identification. rPRVXJ-delgE/gI/TK-S (F5, F10, F15, F20 and F21) TK, gE, gI and S genes were identified by PCR

PCR amplification and sequencing of deleted TK and gE/gI genes and, S genes inserted in the rPRVXJ-delgE/gI/TK-S passaged for 21 generations in BHK-21 cells were performed and it was confirmed that the generated recombinant PRV was genetically stable without any genetic alteration (Fig. 4A-B).

### Safety test of rPRVXJ-delgE/gI/TK-S in mice

**Fig. 5:**
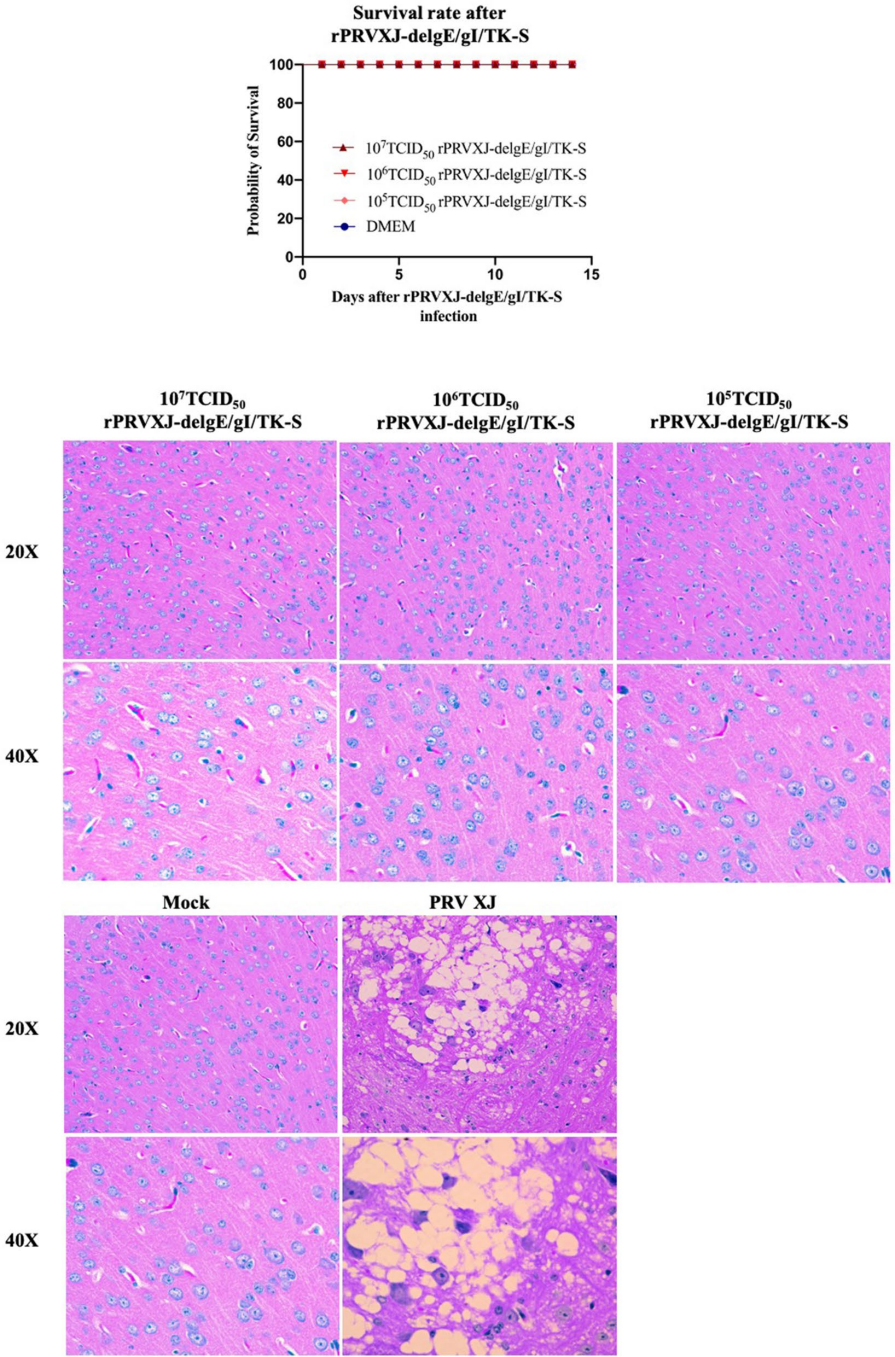
Safety test of rPRVXJ delgE/gI/TK-S in mice. **A**. Safety test of different concentrations (10^7^TCID_50_, 10^6^TCID_50_, 10^5^TCID_50_) of rPRVXJ-delgE/gI/TK-S in mice (*n* = 10/group). **B**. Histopathology examination

After immunizing mice with recombinant virus rPRVXJ-delgE/gI/TK-S, mice did not die (Fig. 5A). Pathology examination showed that PRV XJ strain could cause brain tissue looseness and edema in mice. However, the rPRVXJ-delgE/gI/TK-S did not cause Histological change in mice brain (Fig. 5B). The above data show that the recombinant virus is safe for mice.

### Cellular immunity of mice immunized with recombinant virus rPRVXJ-delgE/gI/TK-S

#### Cytokine expression

**Fig. 6:**
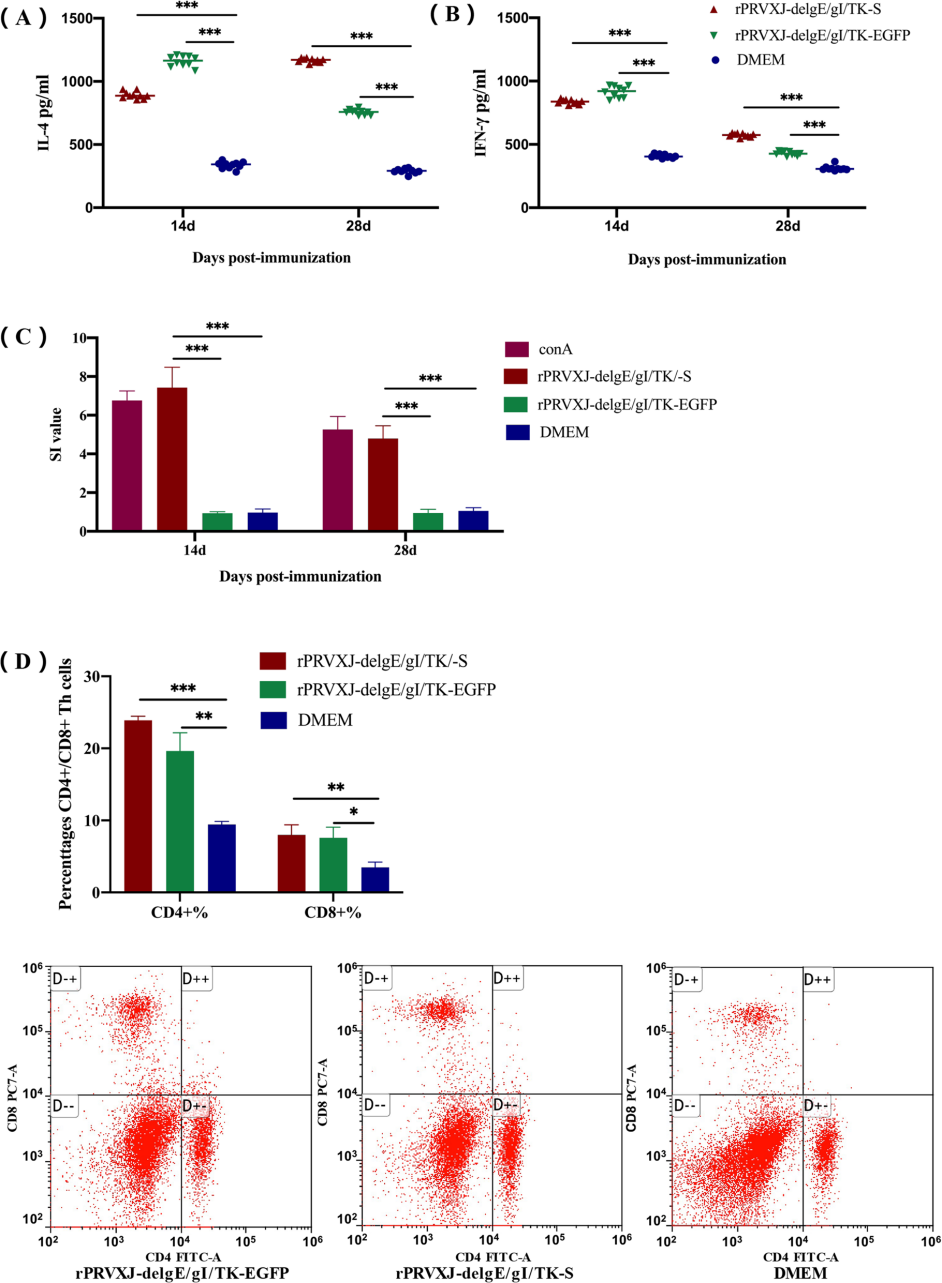
Cell immunity induced by rPRVXJ delgE/gI/TK-S in mice. **A**. Levels of IL-4 secretion. **B**. Levels of IFN-γ secretion. **C**. CCK-8 was used to detect the proliferation of spleen lymphocytes stimulated by PDCoV S protein antigen in each immunized group. **D**. Analysis of CD3+, CD4+ and CD8+ lymphocytes in spleen of mice in each immunization group. Data are expressed as mean ± SD. Significant difference was indicated by “***” (P < 0.001); Extremely significant difference was indicated by “**” (P < 0.01); Significant difference was indicated by “*” (P < 0.05); No significant difference was indicated by “ns” (P > 0.05)

The effective induction of adaptive immunity depends on activation of innate immune system [15], and cytokines can enhance the adaptive immune response by playing a key role in the innate immune response. The levels of Th1 and Th2 cytokines are important indicators of cellular immunity. IFN-γ and IL-4 selected in this study belong to Th1 and Th2 cytokines respectively. The levels of IL-4 and IFN-γ in peripheral blood of mice were detected by cytokine detection kit 14 days and 28 days after immunization. The results showed that the expression levels of IFN-γ and IL-4 in rPRVXJ-delgE/gI/TK-S and rPRVXJ-delgE/gI/TK-EGFP groups were significantly up-regulated compared with DMEM group (Fig. 6A-B).

#### Spleen lymphocyte proliferation test

In order to evaluate the proliferation of T lymphocytes in the spleen of experimental mice, mouse splenocytes were isolated at 14 dpi and 28 dpi respectively, and the proliferation response of specific lymphocytes was measured. When stimulated with purified PDCoV S protein and ConA, the lymphocytes of mice inoculated with rPRVXJ-delgE/gI/TK-S were significantly more than those of mice inoculated with rPRVXJ-delgE/gI/TK-EGFP and DMEM in the group stimulated with purified PDCoV S protein. Their SI values were significantly different (Fig. 6C).

#### Flow cytometry

On the 28th day after immunization, the ratio of CD4^+^/CD8^+^T cells in spleen lymphocytes of mice was measured, and it was found that the ratio of CD4^+^/CD8^+^T cells was different between each group (Fig. 6D). The percentage of CD4^+^ T cells in rPRVXJ-delgE/gI/TK-S and rPRVXJ-delgE/gI/TK-EGFP immunized mice was higher than that in DMEM group, and the percentage of CD8^+^ T cells was higher than that in DMEM group.

### Humoral immunity of mice immunized with recombinant virus rPRVXJ-delgE/gI/TK-S

**Fig. 7:**
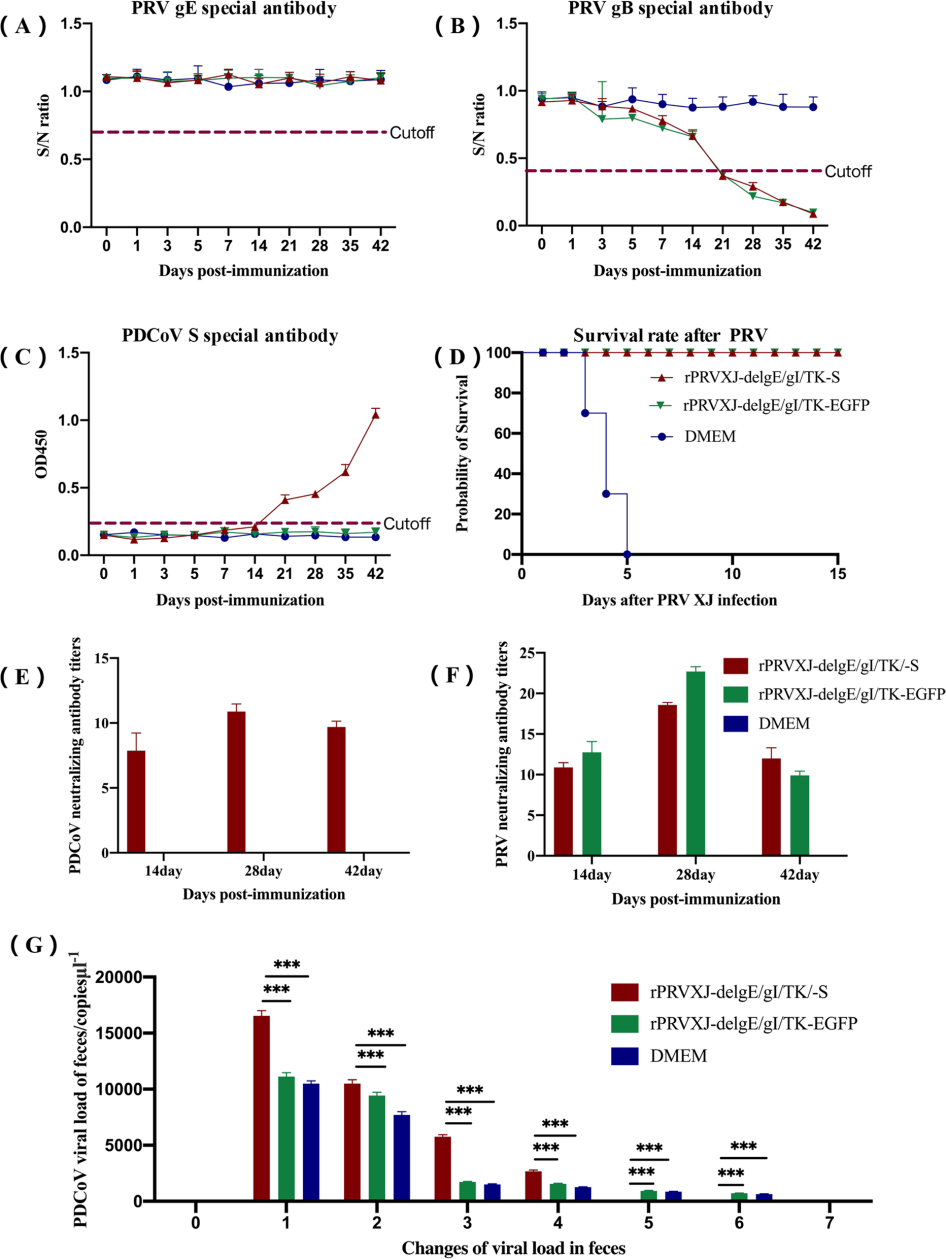
Humoral immunity induced by rPRVXJ delgE/gI/TK-S in mice. **A**. gE specific antibodies. gE specific antibodies were detected by ID.VET blocking ELISA kit. S/N% less than or equal to 60% is positive; S/N% greater than 60% and less than or equal to 70% is suspected; S/N% greater than 70% is negative. **B**. gB specific antibodies. gB specific antibodies were detected by ID.VET blocking ELISA kit. S/N% less than or equal to 40% is positive; S/N% greater than 40% and less than or equal to 50% is suspected; S/N% greater than 50% is negative. **C**. S specific antibodies. The S specific antibody was detected by indirect ELISA method of PDCoV S protein polypeptide antibody constructed in laboratory. OD_450nm_ greater than or equal to 0.233477 was positive; OD_450nm_ less than 0.233477 is negative. **D**. The effectiveness of recombinant virus test. **E**-**F**. Virus neutralization assays. G. PDCoV viral load in feces

To evaluate the humoral immune effect of rPRVXJ-delgE/gI/TK-S in mice, 10^6^TCID_50_ rPRVXJ-delgE/gI/TK-S, 10^6^TCID_50_ rPRVXJ-delgE/gI/TK-EGFP and DMEM were intramuscularly injected with 200 μl, respectively. Two weeks later, a booster immunization program is carried out. Serum samples were collected by caudal vein on day 0, 1, 3, 5, 7, 14, 21, 28, 35, 42, respectively, and the specific antibody levels of gE, gB, and S were measured (Fig. 7A-C).

#### gE-specific antibodies

No PRV-gE-Abs was found in all the experimental and control groups (Fig. 7A).

#### gB-specific antibody

gB antibody detection was positive in rPRVXJ-delgE/gI/TK-S group and rPRVXJ-delgE/gI/TK-EGFP group 1 week after enhanced immunization, and then S/N value gradually increased, and antibody levels in the two groups were similar. PRV-gB-Abs was not observed in the DMEM control group (Fig. 7B).

#### S-specific antibodies

The S-specific antibody in mouse serum was detected by indirect ELISA of PDCoV S protein polypeptide established in the laboratory. One week after booster immunization (the 21st day after the first immunization), S-specific antibody in rPRVXJ-delgE/gI/TK-S group was detected as positive, and then OD_450nm_ value gradually increased. No S-specific antibody was detected in the mice inoculated with rPRVXJ-delgE/gI/TK-EGFP and DMEM during the whole experiment (Fig. 7C).

#### Neutralizing antibody assay

The serum of mice in rPRVXJ-delgE/gI/TK-S group had neutralization effect on PRV and PDCoV, the antibody titers were 1:22.36–1:39.84 and 1:11.22–1:19.96, respectively, reaching the maximum at 28 dpi (Fig. 7E-F).

#### Detoxification of mice

Although there is no literature report that PDCoV can infect mice, we still administered PDCoV disease material to mice to detect the detoxification of mice. The results showed that PDCoV could be detected in the feces of mice collected on the first day, and the viral load was the maximum. After that, it decreased significantly and disappeared gradually. Surprisingly, PDCoV was detected in feces of mice in rPRVXJ-delgE/gI/TK-S group only on days 1–4, while PDCoV was still detected on days 5 and 6 in rPRVXJ-delgE/gI/TK-EGFP group and DMEM group (Fig. 7G). It is suggested that rPRVXJ-delgE/gI/TK-S accelerates the detoxification of PDCoV in mice and may have a protective effect on PDCoV infection, but it needs to be further verified in pigs.

#### Challenge and protection experiment

Six weeks after the first immunization (the 42nd day), each group was challenged with 10 × LD_50_ dose. The mice in the control group began to die on the 3rd day after inoculation with PRV XJ, and all died on the 5th day. Typical neurological symptoms and pruritus appeared before death. rPRVXJ-delgE/gI/TK-EGFP and rPRVXJ-delgE/gI/TK-EGFP immunized mice showed 100% protective effect (Fig. 7D).

## Discussion

The emerging coronavirus could cause widespread transmission among humans and animals, posing a major threat to human health and causing significant economic losses to the global livestock and trade industries. To date, at least six coronaviruses have been found to be pathogenic to pigs, They include porcine epidemic diarrhea virus (PEDV), porcine transmissible gastroenteritis virus (TGEV) and porcine acute diarrhea syndrome coronavirus (SADS-CoV), porcine respiratory coronavirus (PRCV), porcine transmissible gastroenteritis virus (TGEV), porcine haemagglutinative encephalomyelitis virus (PHEV) and porcine deltacoronavirus (PDCoV) [16]. Among them, as PDCoV is a newly emerged enteric pathogenic coronavirus that can cause severe diarrhea of piglets [3] in recent years, few studies have been conducted [17]. Zhao et al. [18] isolated the CHN-SC2015 strain and constructed a phylogenetic tree. The analysis showed that CHN-SC2015 was more closely related to other PDCoV strains in China than the strains from Southeast Asia, the United States, Japan and Korea, in addition,the genetic relationships among these strains were diverse as well as regional and epidemiological characteristics.

Recent studies have shown that the PDCoV strain isolated from China has different degrees of deletion in the Nsp2 and Nsp3 coding regions, and it may have undergone a high degree of mutation, which will lead to increased virulence [19]. According to the data reported by Dong et al. [20], PDCoV existed in China for at least 11 years and could increase the mortality rate of Suckling piglets to more than 80% [21]. In addition, PDCoV can also effectively infect a variety of host cells, including human and chicken, which means that it has a tendency to spread across species [22]. These studies all indicate that persistent PDCoV infections in Asia and North America represent a potential risk of a pandemic outbreak. The prevention and control of PDCoV requires the implementation of effective vaccines. PDCoV is a new enteric pathogenic coronavirus that can cause severe diarrhea in piglets in recent years, but there are few studies on related vaccines at present [23]. However, no commercial vaccine is available, caused a serious problem in controlling the spread of PDCoV.

Coronavirus S protein is the main surface protein and the main target of host humoral immune response, which is thought to be involved in the evolution of the virus and is the focus of vaccine development. Its functions include recognizing receptors and mediating the entry of the virus into host cells, determining the range and tissue tropism of the virus host, and inducing the immune response of the host, etc. [24]. For improving the existing vaccine or developing new vaccine, recombinant virus vaccine is a hot topic in vaccine research and application. The recombinant virus vaccine takes the non-pathogenic or attenuated vaccine virus as the vector, inserts into the immune dominant antigen region of other viruses, modifies the immunogenic protein gene of other pathogens by using the genome of the attenuated vaccine, and directly highly expresses the protein antigen in the host cell. Using the potential adjuvant effect of the virus delivery system itself, it can be directly and effectively delivered to the immune system to produce cellular immunity and humoral immunity [25]. So far, poxviruses, adenoviruses, herpes viruses, reversion-rate viruses, non-segment-specific RNA viruses and so on have been used in the study of live vector vaccines.

Pseudorabies virus (PRV) is a herpesvirus. As a virus vector, almost all foreign gene loci are inserted into TK, gE or gI gene loci in PRV genome. These genes are not necessary for viral replication. When replaced by foreign genes, The virulent strain can be a safe and effective recombinant virus vaccine [26–28]. TK gene is generally the first choice when constructing PRV live attenuated vaccine. It does not participate in virus replication. The deletion of TK gene will reduce the infectivity of the virus to nerve tissue. GE gene is a marker for identifying wild virus and vaccine strain [29].

Diseases caused by PDCoV and PRV are important infectious diseases endangering the global pig industry. They are widespread in many pig farms, which has brought huge economic losses to the pig industry. PDCoV is a new porcine intestinal diarrhea virus emerging in recent years. Up to now, no commercial vaccine has been used to prevent and control this disease. With the further research on the pathogenesis and immune mechanism of PDCoV, commercialized vaccines and diagnostic reagents will be available in the future to control the occurrence and prevalence of the disease.

In this study, we constructed recombinant pseudorabies strain rPRVXJ-delgE/gI/TK-S expressing PDCoV S protein, studied some biological characteristics of the recombinant virus, such as genetic stability, one-step growth curve, virus particle size and plaque size, and evaluated the ability of the recombinant virus to induce humoral and cellular immunity in mice. First, gene stability is important for live vector vaccine strains. The recombinant strain rPRVXJ-delgE/gI/TK-S constructed in this study stablely deleted PRV gE, gI TK genes, and inserted PDCoV S gene, and no mutation was observed when passed to the 21st generation. Compared with the parent strain PRV XJ and the three-gene deletion strain rPRVXJ-delgE/gI/TK-EGFP, the insertion of S gene reduced the virus titer and plaque size but had little effect on the growth characteristics. Secondly, the ideal vaccine vector must be safe in general, and the inserted exogenous gene should not limit the immunogenicity or growth characteristics compared with the parent virus [32]. The results of this study showed that the inoculation of rPRVXJ-delgE/gI/TK-S did not cause death in mice and had good immunogenicity. In the aspect of humoral immunity, rPRVXJ-delgE/gI/TK-S caused mice to produce gB and S-specific antibodies, did not produce gE antibodies, and could also cause mice to produce neutralizing antibodies. In terms of cellular immunity, both the recombinant strain and the three gene deletions strain could induce up-regulation of Th1 cytokines IFN-γ and Th2 cytokines IL-4. Only spleen lymphocytes of mice immunized with rPRV XJ-delgE/gI/TK-S could proliferate under the stimulation of PDCoV S protein (10 μg/mL). The results of flow cytometry showed that compared with mice inoculated with DMEM, rPRVXJ-delgE/gI/TK-S could increase the percentage of CD4+ and CD8 + T cells in spleen. Finally, in the face of the attack of the parent strain PRV XJ, it has 100% protective ability against mice and speeds up the excretion of PDCoV in mice, but the protective effect on PDCoV needs to be further verified in pigs.

## Conclusion

In summary, we constructed recombinant virus rPRVXJ-delgE/gI/TK-S expressing PDCoV S protein using CRISPR/Cas9 gene editing technology and homologous recombination technology. The recombinant virus showed good safety and immunogenicity in mice and could induce the production of high-level antibodies. It has 100% protection against parental strain PRV XJ attack. Therefore, it can be used as a candidate recombinant vaccine for the prevention of PDCoV and pseudorabies, but further research is needed to confirm the effectiveness of rPRVXJ-delgE/gI/TK-S in pigs.

## Materials and methods

### Cells, viruses, disease material, plasmid and polyclonal antibody

rPRVXJ-delgE/gI/TK-EGFP and rPRVXJ-delTK strains were constructed and preserved by Animal Biotechnology Center, College of Veterinary Medicine, Sichuan Agricultural University (Chengdu, China), and proliferated on BHK-21 cells. BHK-21 cells were cultured in DMEM (Gibco) supplemented with 10% newborn bovine serum (BI) (37 °C, 5% CO_2_); PDCoV positive fecal materials were identified and preserved by Sichuan Agricultural University; CRISPR/Cas gE, pEGFP-gI28K eukaryotic expression plasmid containing homologous arm of pseudorabies gI and 28 K gene sequence and positive plasmid standard pMD-S (PDCoV) were constructed by Animal Biotechnology Center of Sichuan Agricultural University. PDCoV S protein and its murine polyclonal antibody were prepared and preserved by Animal Biotechnology Center of Sichuan Agricultural University.

### Construction of recombinant transfer plasmids

We designed and synthesized two pairs of specific primers for amplification of PDCoV S gene (2401-3300 bp, encoding 800-1100aa) based on the strains PDCoV CH/Sichuan/S27/2012 in GenBank (GenBank No.: KT266822.1), then, introduced EcoRI and MluI ribozyme restriction sites and the homologous arm of pEGFP-gI28K vector (Table 1) at Shanghai Sangon Biotechnology Co., Ltd. We amplified the target sequence by the laboratory preserved PDCoV disease material. The eukaryotic transfer vector pEGFP-gI28K-PDCoV-S was constructed using the Seamless Cloning Kit, and the Plasmid was extracted according to the instructions of the End-free Plasmid Mini Kit II (Omega). The correctness of the method was verified by enzyme digestion and sequencing (data not shown).

**Table 1:** Sequences of oligonucleotides used for PCR in this study

Name	Sequence (5′-3′)	Purpose
gE	ATCTGGACGTTCCTGCCC	Detecting PRV gE gene Amplifying the TK gene of PRV
	GTAGATGCAGGGCTCGTACA	
TK	GATGACATACACATGGCTTTATACGCGCC	
	TCACCGCCGCGGCCCGGCGACGTACTC	
gI	TCGCCGAGCAACTACAGCGG CGGCGTCGTCGTCTCCGCGT	Detecting PRV gI gene Amplifying the S gene of PDCoV
S	CCAGCAAGTTGACCGTCTCA	
	CCATTTGGTGCAGTCTGTGT	
pMD-S	TTGTTAACCAGCAGGGCGAG AGCCAACCGTCCTGTGATG	Detection PDCoV S gene for qPCR
pEGFP-gI28k-PDCoV-S	TCGAGCTCAAGCTTC***GAATTC***CAGCAAACTTCTGAGGCTCTT	Construction of recombinant transfer plasmids
	CGAGCCGGGGGAGAT***ACGCGT***ATACCACGGCCGTTTAAGGTAAGT	

### Construction of the recombinant virus rPRVXJ delgE/gI/TK-S

Refer to the instructions of LipofectamineTM 3000 Reagent (Invitrogen, USA) for transfection kit. CRISPR-Cas gE and pEGFP-gI28K-PDCoV-S were co-transfected into BHK-21 cells. After 24 h, 10 μl rPRVXJ-delTK virus was added. When 80% of the lesions appeared, the cells were freeze-thawed for 3 times in a refrigerator at − 80 °C to collect the disease venom. The recombinant virus was purified by 96-well plate limited dilution method and 6-well plate virus plaque purification method.

### Verification of recombinant virus rPRVXJ delgE/gI/TK-S

#### PCR

Specific primers (Table 1) were used to analyze the TK, gE and gI genes missing in rPRVXJ-delgE/gI/TK-S DNA, and the S genes inserted were respectively analyzed. PRV XJ DNA was used as the positive control, and the template without DNA was used as the mock control.

#### Immunofluorescence assay

rPRVXJ-delgE/gI/TK-S, rPRVXJ-delgE/gI/TK-EGFP and PRV XJ 5 μl were inoculated when BHK-21 cells grew to a dense monolayer on the 24-well plate. When the cells were diseased but not completely detached, the supernate was discarded, fixed with 4% paraformaldehyde for 30 min, permeated with 30% acetone for 20 min, and sealed with 5%BSA for 60 min. The cells were incubated overnight with polyclonal anti-S mouse antibody (1:200) and incubated for 2 h with FITC-conjugated goat anti-mouse IgG (1:300) for primary and secondary antibodies, respectively. Note that the cells need to be washed with PBS for 3 times in each step, 5 min each time. Fluorescence lesions were observed under a fluorescence microscope.

#### Western blot analysis

The recombinant viruses rPRVXJ-delgE/gI/TK-S, rPRVXJ-delgE/gI/TK-EGFP and PRV XJ were infected with BHK-21 cells, and the cells were harvested with RIPA containing 1 mM phenylmethane sulfonyl fluoride (PMSF) (Biyuntian). After protein extraction, the protein was separated by 12% SDS-PAGE and transferred to nitrocellulose membrane. At room temperature, it was sealed with 5% skim milk (prepared by PBS) for 2 h. After being washed with PBST (0.5% twain-20 in PBS), it was incubated with primary antibody (1:200 diluted polyclonal anti-S mouse antibody and 1:5000 diluted reference gene GAPDH) at 4 °C for 24 h. After washing, the secondary antibody (horseradish peroxidase (HRP) -bound IgG antibody) (ABCAM, No.: AB170487) was diluted at 1:5000 and incubated at room temperature for 2 h. Imaging with hypersignal detection of protein bands by the West Pico Plus Chemiluminescence Branch (Thermosciences, USA) and Chemiluminescence Imaging System (Chemidoc MP Bio-Rad, CA, USA).

### Biological characteristics of recombinant virus rPRVXJ delgE/gI/TK-S

#### Plaque assays

BHK-21 cells were passaged to 6-well plates to make the cell density about 2 × 10^6^–4 × 10^6^/ml. After gradient dilution (10^− 2^–10^− 7^), the recombinant virus rPRVXJ-delgE/gI/TK-S, the gene deletion strain rPRVXJ-delgE/gI/TK-EGFP, and the parent strain PRV XJ were inoculated with 200 μl virus venomous in each well, and placed in a 37 °C cell incubator with 5% CO_2_ for adsorption for 1 h. The liquid in the wells was then discarded and washed twice with PBS. The mixture of 1% methyl cellulose and 2 × DMEM1:1 was mixed and covered with 2 ml of each well. After incubation at 37 °C for 48 h, the excess crystal violet was stained with 5% (W/V) for 30 min, and the superfluous crystal violet was washed with distilled water. The morphology of virus plaques formed by the three strains were observed and photos were collected.

#### Growth kinetics

In order to analyze the growth characteristics of the recombinant virus, BHK-21 cells cultured in 12-well were infected with 10^4^TCID_50_ rPRVXJ-delgE/gI/TK-S, rPRVXJ-delgE/gI/TK-EGFP and PRV XJ, 100 μl per well. The cells were adsorbed in an incubator at 37 °C and 5%CO_2_ for 1 h, and then the cells were washed with PBS for 3 times. Then 1 ml DMEM cell maintenance solution was added into each well, and the maintenance solution was added for 0 h. All cells and supernatants were collected at 0 h, 2 h, 4 h, 6 h, 8 h, 10 h, 12 h, 24 h, 36 h, 48 h, 60 h and 72 h, and 3 samples were taken at each time point as replicates. The 50% tissue culture infection dose (TCID_50_) was calculated by Reed-Muench method, and the one-step growth curve of the virus was plotted.

#### Electron microscopy analysis

BHK-21 cells were subcultured into 6-well plates, and when the concentration of cells reached 80%, 10^4^TCID_50_ dose of rPRVXJ-delgE/gI/TK-S was inoculated into each well, and the cells were placed in a 5% CO_2_ cell incubator at 37 °C for adsorption for 1 h. The liquid in the hole was then discarded and 2 ml DMEM containing 2% FBS was added. After 24 h, the cell samples in the well were collected, treated with 0.5% glutaraldehyde fixed solution and 3% glutaraldehyde fixed solution, and then sent to Chengdu Lilai Biotechnology Co., Ltd. (photos of virions were collected by JEM-1400plus transmission electron microscope).

#### Genetic stability

To evaluate the genetic stability, the recombinant virus rPRVXJ-delgE/gI/TK-S was passaged 21 times in BHK-21 cells. The deletion of TK, gE and gI genes and insertion of S genes were verified by PCR in F5, F10, F15, F20 and F21. At the same time, the expression of S gene was confirmed by IFA with mouse PDCoV S protein polyclonal antibody. PRV XJ DNA was used as the positive control, and the template without DNA was used as the negative control.

### Experimental infections of mice

#### Safety test of rPRVXJ delgE/gI/TK-S in mice

10^7^TCID_50_,10^6^TCID_50_,10^5^TCID_50_rPRVXJ-delgE/gI/TK-S and DMEM were injected intramuscularly, each 200 μl. The survival of mice was recorded and the safety of recombinant virus to mice was evaluated.

After 15 days, the brain tissue of mice was fixed with 4% paraformaldehyde and sent to Wuhan Servicebio for pathological examination.

#### Immunogenicity of rPRVXJ delgE/gI/TK-S in mice

All the animal experiments had been approved by the Laboratory Animal Management Committee of Sichuan Province (Approval Number SYXK2019–187).

Female BALB/c mice aged 6–8 weeks were purchased from Chengdu Dashuo Biotechnology Co., Ltd. (Chengdu, China). The mice were randomly divided into three groups (30 mice in each group). The mice were intramuscular injected with 10^6^TCID_50_ rPRVXJ-delgE/gI/TK-S, 10^6^TCID_50_ rPRVXJ-delgE/gI/TK-EGFP and DMEM 200 μl. Intensify immunization after two weeks. Blood was collected through the tail vein on days 0, 1, 3, 5, 7, 14, 21, 28, 35 and 42 respectively, and mouse splenocytes were isolated on days 14 and 28.

Another independent experiment: mice were randomly divided into four groups (rPRVXJ-delgE/gI/TK-S immune group, rPRVXJ-delgE/gI/TK-EGFP immune group and two DMEM groups), with 10 mice in each group. After 6 weeks of intensify immunization, rPRVXJ-delgE/gI/TK-S immunization group, rPRVXJ-delgE/gI/TK-EGFP immunization group and DMEM group were injected intramuscularly for 10 × LD_50_ dose of PRV XJ. The survival of mice was recorded. After 14 days, all mice were fed with 150 μl PDCoV disease material, and mouse feces were collected on days 0, 1, 2, 3, 4, 5, 6 and 7.

The LD_50_ of BALB/c mice was determined by Reed-Muench/Karber method.

### Neutralizing antibody assay

The collected mouse serum (14 dpi, 28 dpi, 42 dpi) was inactivated by water bath at 56 °C for 30 min. After continuous double dilution (1:2n) with DMEM, it was mixed with 200TCID_50_ virus solution, and incubated in 5% CO_2_ incubator at 37 °C for 1 h. When the cells grew to a dense monolayer, the mixture was added and cultured in a 5% CO_2_ incubator at 37 °C, and CPE was recorded for 5–7 days. The average neutralizing antibody titer of three measurements was calculated according to Reed-Muench method.

### Enzyme-linked immunosorbent assay (ELISA)

#### gB 、gE-specific antibodies and cytokine detection

According to the manufacturer’s instructions, the commercial gE, gB ELISA Kit (ID.VET, Innovactive Diagnostics, French) and cytokine detection kit (Neobioscience, China) were used to detect PRV specific gE, gB antibody, IL-4 and IFN-γ on days 0, 1, 3, 5, 7, 14, 21, 28, 35 and 42 after vaccination.

#### PDCoV S-specific antibodies detection

Serum S-specific antibodies in each group were detected at 0, 1, 3, 5, 7, 14, 21, 28, 35, 42 days after immunization. The screened S protein polypeptide VTPRNMYEPRLPRQA was used as the coating antigen, the polypeptide protein was diluted to 500 ng/ml with coating solution, and then coated with ELISA plate, with100 μl/well at 37 °C for 2 h. The coated solution was discarded and washed with 200 μl PBST for 3 times, 5 min/ time. 200 μl 5% skimmed milk in PBS/well at 37 °C for 2 h, then the blocking liquid was discarded and washed with 200 μl PBST for 3 times, 5 min/time. Then the collected mouse serum was diluted with PBS at the ratio of 1:1, and the positive serum control (murine polyclonal antibody of S protein) and negative serum control were set at the same time. The mice serum was incubated at 37 °C for 30 min at 100 μl/well, then the mouse serum was discarded and washed with 200 μl PBST for 3 times, 5 min/time. HRP-labeled goat anti-mouse IgG (1:3000 dilution) was diluted with PBS at 100 μl/well and incubated at 37 °C for 30 min. The secondary antibody was discarded and washed with 200 μl PBST for 3 times, 5 min/time. Add 100 μl TMB substrate chromodeveloping solution to each well, and incubate at 37 °C for 15 min. Finally, 50 μl 2MH_2_SO_4_ was added to each well to terminate the reaction. OD_450_nm absorbance value was detected by microplate analyzer within 15 min.

### Spleen lymphocyte proliferation test

On day 14 and 28 after infection, splenic cells were isolated in 1640 medium, and were spread on 96-well plates (100 μl per well) with the number of 5 × 10^6^ cells/ml. The proliferation of spleen cells in each mouse was measured under three conditions:(1) purified PDCoV S protein (10 μg/ ml); (2) ConA (10 μg/ ml); (3) 1640 medium. After 72 h of culture, the OD_450nm_ absorbance value of each well was detected by CCK-8 (Beyotime, China) detection kit and the stimulation index was calculated to evaluate the proliferation of splenic lymphocytes. SI = (OD values of immunized groups-OD values of blank control)/(OD values of negative control group-OD values of blank control).

### Analysis of CD3+, CD4+ and CD8+ T lymphocytes

On day 14 after enhanced immunization, spleen cells were isolated from each group and stained with PE-CyTM rat anti-mouse CD8a (BD Pharmingen), FITC rat anti-mouse CD4 (BD Pharmingen), APC Hamster Anti-Mouse CD3e (BD Biosciences Pharmingen) at room temperature for 30 min in dark, then detected by flow cytometry (Backman, USA). The data were analyzed using Kaluza 2.1 software.

### Detoxification of mice

The detoxification of mice was detected by real-time fluorescence quantitative PCR of PDCoV S gene established in the laboratory. Primers were designed using Oligo 6.0 software and are shown in Table 1.Extract the total RNA of feces collected in 2.10.2 according to the instructions of trizolrna extraction kit (Takara), according to the manufacturer’s instructions and the RNA concentration was measured using a nucleic acid concentration analyzer (Scandrop200, Analytik Jena, Germany). Reverse transcription was performed using the Prime Script™ II 1st Strand cDNA Synthesis Kit (Takara), and qPCR was performed in a Light Cycler 96 (Roche, Switzerland) with TB Green® Premix Ex Taq™ II (Tli RNaseH Plus) (Takara). The thermal cycling conditions were 95 °C for 30 s, followed by 40 cycles of 95 °C for 5 s, 60 °C for 30 s, and 72 °C for 30s.

### Statistical analysis

Statistical analysis was performed and histogram were drawn using GraphPad PrismTM 8.0 (GraphPad Software, USA), Paired student *t*-test, and one-way ANOVA was used to test differences between different groups. *P* values< 0.05 were considered significant.

## Supplementary Information

**Additional file 1.**

## Data Availability

The raw data is available from the corresponding author upon reasonable request.

## Abbreviations

PDCoV: Porcine deltacoronavirus
S protein: Spike protein
BHK-21: Baby hamster syrian kidney-21
IFN-γ: Interferons-γ
IL-4: Interleukin-4
CD3 +: Cluster of differentiation 3+
CD4 +: Cluster of differentiation 4+
CD8 +: Cluster of differentiation 8+
Dpi: Days post- immunization
PRV: Pseudorabies virus
PEDV: Porcine epidemic diarrhea virus
TGEV: Porcine transmissible gastroenteritis virus
PRCV: Porcine respiratory coronavirus
PHEV: Porcine haemagglutinative encephalomyelitis virus
PMSF: Phenylmethane sulfonyl fluoride
TCID50: 50% tissue culture infective dose
ELISA: Enzyme linked immunosorbent assay
CCK-8: Cell Counting Kit-8
SI: Stimulation index
DMEM: Dulbecco’s Modified Eagle’s medium
FBS: Foetal bovine serum
qPCR: Real-time polymerase Chain Reaction
PBST: 0.5% Tween-20 in PBS
HRP: Horseradish peroxidase
IgG: Immunoglobulin G
CPE: Cytopathic effect
PCR: Polymerase chain reaction
IFA: Indirect immunofluorescence
