# Supplementary figures and images for: The recombinant pseudorabies virus expressing porcine deltacoronavirus spike protein is safe and effective for mice
Source: BMC Vet Res. 2022 Jan 4;18:16. doi: 10.1186/s12917-021-03115-1 (PMC8725529; doi:10.1186/s12917-021-03115-1)

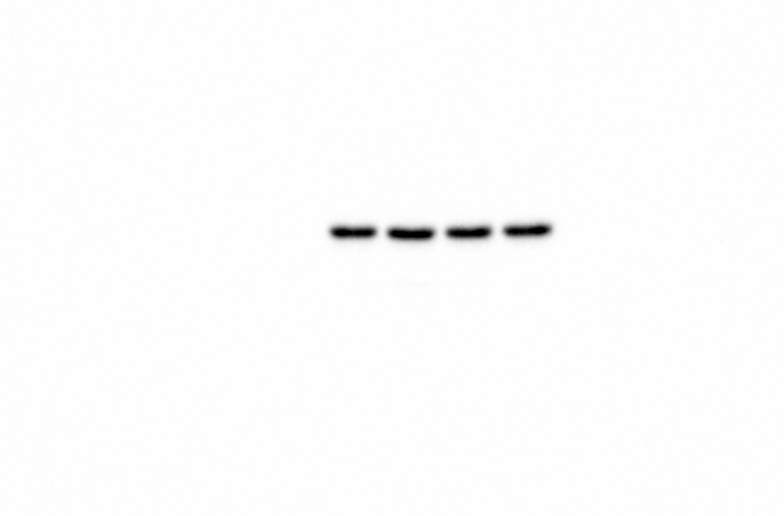

Supplement: Supplementary file 1 — Additional file 1. [file 12917_2021_3115_MOESM1_ESM.zip › GAPDH.tif]

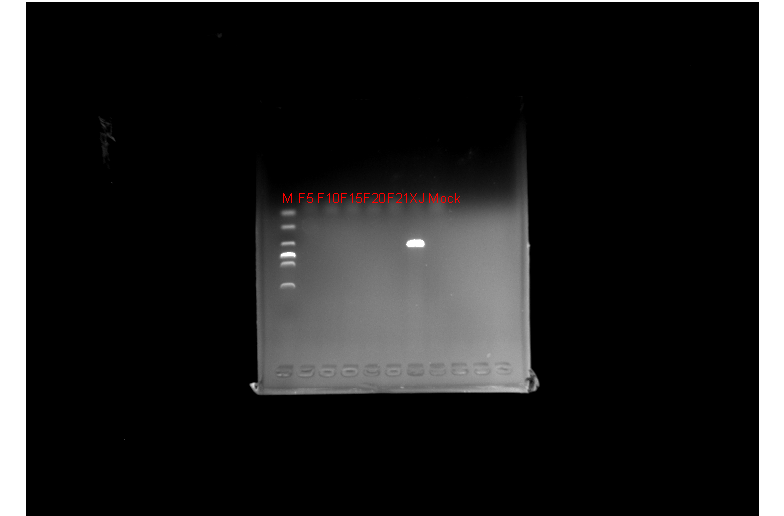

Supplement: Supplementary file 1 — Additional file 1. [file 12917_2021_3115_MOESM1_ESM.zip › gE gene.tif]

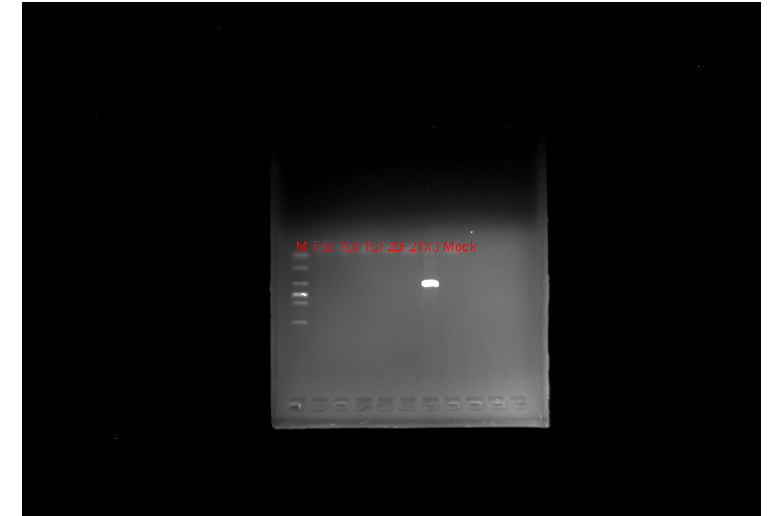

Supplement: Supplementary file 1 — Additional file 1. [file 12917_2021_3115_MOESM1_ESM.zip › gI gene.tif]

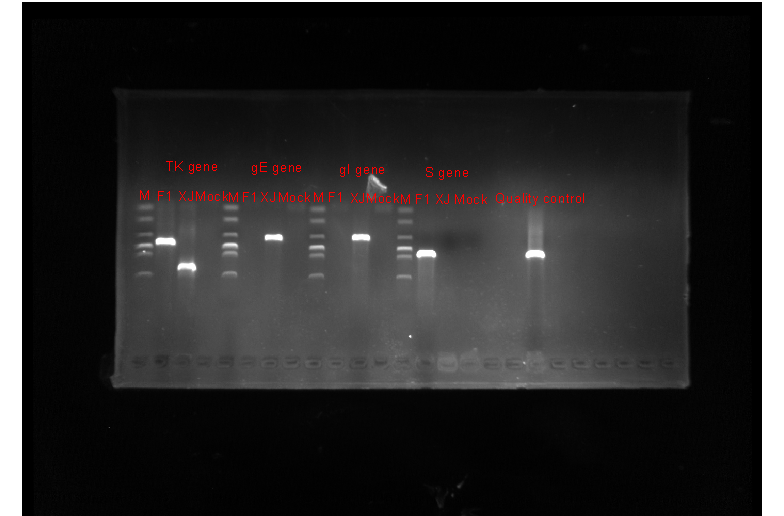

Supplement: Supplementary file 1 — Additional file 1. [file 12917_2021_3115_MOESM1_ESM.zip › PCR analysis of deletion_insertion genes.tif]

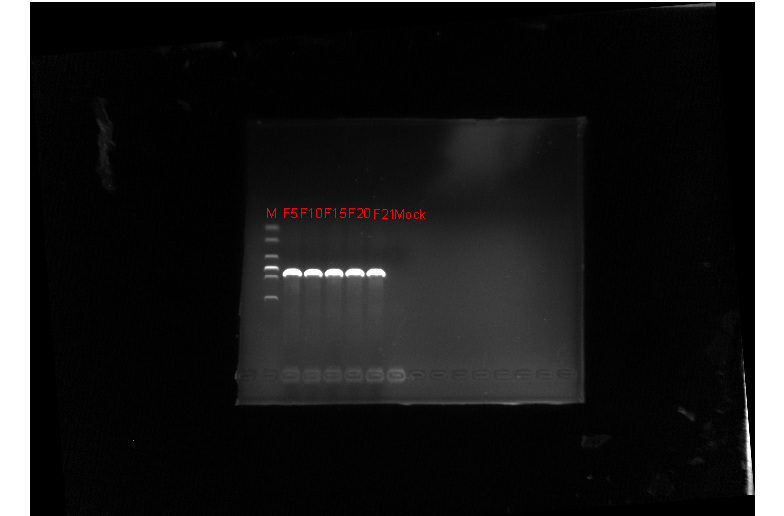

Supplement: Supplementary file 1 — Additional file 1. [file 12917_2021_3115_MOESM1_ESM.zip › S gene.tif]

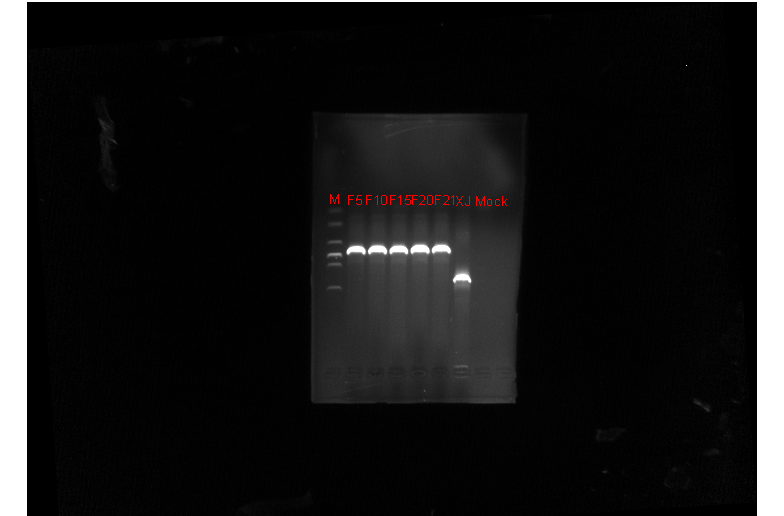

Supplement: Supplementary file 1 — Additional file 1. [file 12917_2021_3115_MOESM1_ESM.zip › TK gene.tif]

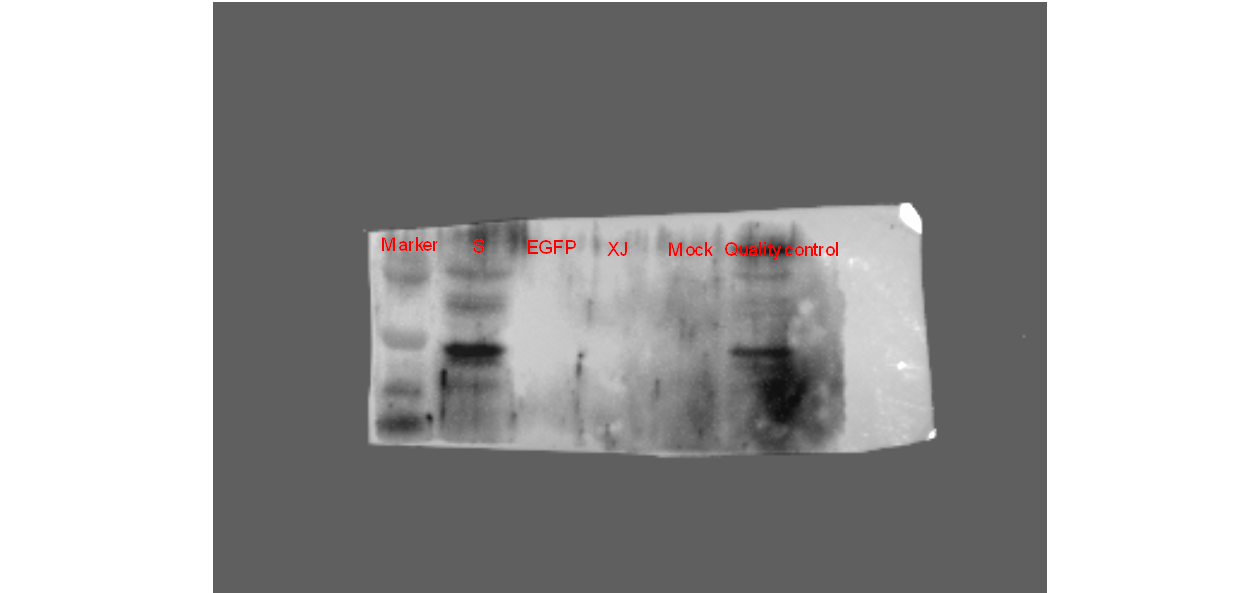

Supplement: Supplementary file 1 — Additional file 1. [file 12917_2021_3115_MOESM1_ESM.zip › wb.tif]
